# Personalized Treatment Recommendation System in Head and Neck Cancer Using Survival Analysis and Deep Learning

**DOI:** 10.3390/healthcare14152275

**Published:** 2026-07-25

**Authors:** Xijing Fei, Kai Liu, Narayanaswamy Balakrishnan

**Affiliations:** 1 School of Mathematics and Science, Xi’an Jiaotong-Liverpool University, Suzhou 215123, China; xijing.fei24@student.xjtlu.edu.cn; 2 School of Statistics and Mathematics, Interdisciplinary Research Institute of Data Science, Shanghai Lixin University of Accounting and Finance, Shanghai 201209, China; 3 Department of Mathematics and Statistics, McMaster University, Hamilton, ON L8S4K1, Canada; bala@mcmaster.ca; 4 Department of Mathematics, Atilim University, Ankara 06836, Turkey

**Keywords:** survival analysis, personalized medicine, deep learning, treatment recommendation, clinical decision support

## Abstract

**Background/Objectives**: Individualized treatment selection for head and neck cancer requires survival models that use routine clinical variables while accounting for censored time-to-event outcomes. This study developed an interpretable Cox proportional hazards baseline and a DeepSurv framework to estimate mortality risk and explore treatment-specific predictions among radiotherapy-based options. **Methods**: Clinical data were obtained from the RADCURE collection in The Cancer Imaging Archive. After preprocessing, stage harmonization, exclusion of sparse treatment categories, missing-data assessment, one-hot encoding, and standardization, 3266 patients were analyzed. Cox regression and DeepSurv were evaluated using a held-out 80%/20% split, paired bootstrap confidence intervals for C-index differences, and five-fold cross-validation. For treatment recommendation, treatment modality was hypothetically varied across radiotherapy alone, chemoradiotherapy, and radiotherapy plus epidermal growth factor receptor inhibitor while other covariates were held fixed. **Results**: On the held-out test set, Cox achieved a C-index of 0.684 and DeepSurv achieved a C-index of 0.695. The absolute difference was 0.011, with a paired bootstrap 95% CI of −0.010 to 0.030, indicating no statistically significant improvement. Five-fold cross-validation showed mean C-index values of 0.688 for Cox and 0.707 for DeepSurv. Cox regression identified older age and advanced tumor stage as higher-risk factors, whereas former and non-smoking status were associated with lower hazard than current smoking. **Conclusions**: DeepSurv provided only a modest numerical gain over the Cox baseline. The recommendation framework illustrates how survival models can generate treatment-specific risk estimates, but these outputs should be interpreted as decision-support signals rather than causal treatment effects. External validation and prospective evaluation are needed before clinical use.

## 1. Introduction

Precision medicine has become an important direction in modern healthcare [1]. Its goal is to support individualized clinical decisions based on patient characteristics [2]. In diseases such as cancer, survival outcomes often vary substantially across patient subgroups, making prognosis estimation and treatment selection particularly important [3]. This study focuses on developing an individualized treatment recommendation system for head and neck cancer based on survival analysis models.

Previous studies of head and neck cancer have applied machine learning and deep learning approaches to survival prediction and clinical decision support. Han et al. [4] demonstrated the feasibility of machine-learning-based survival prediction in head and neck squamous cell carcinoma using approaches including random forests, support vector machines, and DeepSurv. However, their primary objective was prognostic modeling rather than individualized treatment recommendation. Howard et al. [5] developed a machine learning framework to guide adjuvant treatment decisions and evaluate treatment outcomes, although their clinical decision setting and modeling strategy differed from the radiotherapy-based treatment scenarios considered in the present study. Alabi et al. [6] developed and externally validated machine-learning models for survival prediction in head and neck squamous cell carcinoma using multicenter datasets, highlighting the importance of model generalizability and explainability for clinical implementation. Araujo et al. [7] systematically reviewed machine learning approaches for predicting treatment-related toxicities, while Moharrami et al. [8] reviewed machine learning models for outcome prediction in head and neck cancer. Concha Fernández et al. [9] summarized emerging applications of artificial intelligence for risk stratification in head and neck cancer and emphasized the need for robust validation, model interpretability, and responsible clinical implementation. These studies and reviews highlighted the increasing application of machine learning in oncology but also emphasized heterogeneity in data sources, clinical endpoints, and validation strategies. Although these studies demonstrated promising predictive performance, most focused on prognosis estimation, treatment outcome assessment, or toxicity prediction rather than survival-analysis-based individualized treatment recommendation.

Many classification methods simplify outcomes into binary categories, such as alive versus dead, without fully accounting for censoring or time-to-event information [10]. Survival analysis methods address these limitations by incorporating censored observations, estimating risk over time, and generating survival curves [11]. Among these approaches, the Cox proportional hazards model is one of the most widely used methods in medical statistics because it relates covariates to hazard rates without specifying the baseline hazard function [12]. Its interpretability and flexibility make it valuable for clinical decision support.

However, conventional Cox regression primarily provides population-level hazard ratios (HRs) rather than individualized treatment recommendations. For example, a Cox model may show that one treatment is associated with a lower overall hazard of death than another, but it does not directly determine the best treatment for a specific patient with unique characteristics such as age, smoking history, or tumor stage. Personalized recommendation therefore requires estimating survival outcomes for the same patient under multiple treatment scenarios [13].

The main objectives of this study were to build a reliable baseline survival model using a Cox model, evaluate model assumptions and predictive performance, and develop an individualized treatment recommendation framework. In addition to classical survival modeling, this study explores machine learning approaches such as DeepSurv, which extends the Cox model through neural networks to capture nonlinear relationships between covariates [13].

In this context, the study by Zhang et al. [14] represents the closest related work, as it also developed a deep-learning-based framework for individualized treatment recommendation in head and neck cancer. Their study focused on recommendations among major treatment strategies, including surgery, chemoradiotherapy, and postoperative chemoradiotherapy, and further evaluated survival differences between patients whose treatments were consistent or inconsistent with model recommendations. In contrast, the present study focuses on treatment selection among radiotherapy-based treatment options, including radiotherapy (RT) alone, chemoradiotherapy (ChemoRT), and radiotherapy combined with an epidermal growth factor receptor inhibitor (RT + EGFRI). Rather than comparing broad treatment strategies, this study aims to investigate whether survival prediction models can be used to support individualized treatment recommendations within radiotherapy-based clinical decision-making. To achieve this, the study develops a survival prediction framework based on routinely available clinical variables to estimate patient-specific survival risk under alternative treatment options. Overall, this work seeks to explore the potential application of survival modeling and machine learning in precision medicine for personalized treatment recommendation [15,16].

## 2. Data Source and Original Variables

The dataset used in this work was obtained from the RADCURE collection available through The Cancer Imaging Archive (TCIA). The RADCURE dataset contains clinical information for patients with head and neck cancer treated at the University Health Network (UHN) in Toronto, Canada, between 2005 and 2017. The original dataset included 3346 patients and 34 clinical variables related to demographic characteristics, smoking history, disease site and stage, pathology, HPV status, treatment delivery, follow-up, death, recurrence, second cancer, dataset partition, and imaging contrast status. Table 1 provides a description of all 34 original variables and their coding or observed categories where applicable.

**Figure 1:**
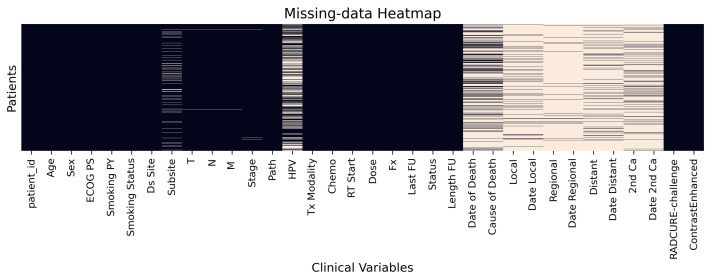
Missing-data heatmap. Black cells indicate observed values, whereas light beige cells indicate missing values.

Missingness was assessed both visually and numerically. Figure 1 shows the overall missing-data pattern, and Table 2 summarizes the missing-data rates for variables with at least one missing value in the original dataset.

## 3. Methodology

**Figure 2:**
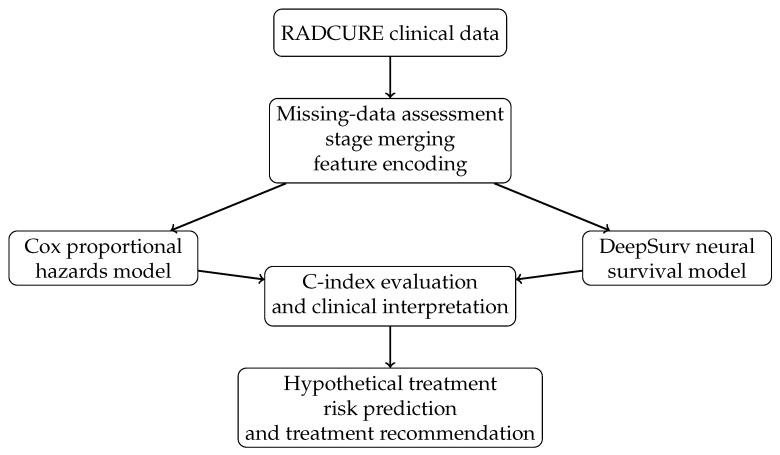
Overview of the proposed survival-modeling and treatment-recommendation workflow.

The analysis proceeded from preprocessing and feature engineering to survival-model development, model evaluation, and exploratory treatment-specific risk prediction. The overall workflow is shown in Figure 2.

### 3.1. Preprocessing, Variable Selection, and Feature Engineering

Before model development, the original RADCURE clinical variables were reviewed to define baseline predictors, survival outcomes, and the analysis cohort. The survival outcome was defined using Length FU, representing follow-up duration in years, and Status, representing each patient’s vital status at the last follow-up. Patients labeled as Dead were treated as events, whereas patients labeled as Alive were treated as right-censored observations.

Candidate baseline predictors were selected according to missingness, timing, clinical relevance, and redundancy. Subsite was not retained as a primary predictor because it had non-negligible missingness and high categorical cardinality, with 63 observed labels, which would have introduced sparse one-hot encoded features relative to the available sample size and reduced model stability. HPV status was also not retained as a primary predictor because it had substantial missingness, with 1629 missing values, corresponding to 48.7% of the original cohort. Including HPV status in the primary model would therefore have required either excluding a large number of patients or performing extensive imputation for a variable missing in nearly half of the cohort, which could introduce additional uncertainty. Variables describing post-baseline outcomes or follow-up events, including Date of Death, Cause of Death, Local, Date Local, Regional, Date Regional, Distant, Date Distant, 2nd Ca, and Date 2nd Ca, were excluded from the predictor set to avoid information leakage. RADCURE-challenge was not used because it represents the original challenge split. Instead, this study used the full eligible sample with a new random 80%/20% train-test split and five-fold cross-validation. ContrastEnhanced was not included because it is an imaging acquisition variable rather than a structured clinical predictor in the current model. RT Start, Dose, Fx, and Last FU were also not used as baseline predictors because they represent treatment delivery or follow-up information rather than pretreatment clinical characteristics.

To reduce redundancy, Tx Modality was retained as the treatment strategy variable, whereas Chemo was excluded because it overlapped substantially with treatment modality. Similarly, the overall tumor stage was retained and harmonized as the stage predictor because it summarizes the information contained in the T, N, and M classifications; the individual T, N, and M variables were not included in the primary model to avoid redundant information and potential multicollinearity.

Sample selection and category harmonization were then performed to improve model stability. The postoperative RT alone group was removed because it contained only three patients, which was insufficient for meaningful survival modeling and treatment comparison. Tumor stage categories were merged to reduce sparsity and improve interpretability. Specifically, stage IA and IB were combined into stage I, while stage IIA and IIB were combined into stage II. Advanced stages were similarly grouped into broader stage categories. Patients with unknown or missing stage information, including empty values, X, and 0, were excluded from the analysis.

After the analysis variables were defined, categorical predictors, including sex, smoking status, merged stage, and treatment modality, were converted into numerical form using one-hot encoding [17]. To avoid multicollinearity, one category from each variable was selected as the reference group and omitted during encoding [18]. Continuous predictors, including age and smoking pack-years, were standardized using z-score normalization [17]. Standardization centers variables at a mean of zero and scales them to a standard deviation of one, which is especially important for neural-network training stability [19]. Data processing was conducted in Python 3.12.3 using pandas and NumPy, and data splitting and standardization used scikit-learn. For held-out testing and cross-validation, encoding and scaling were fitted within the training data or training fold and then applied to the corresponding test or validation data to reduce data leakage. A fixed random seed was used for the 80%/20% train-test split to improve reproducibility.

The formal statistical analysis began with a classical Cox proportional hazards model and then progressed toward individualized treatment recommendation using DeepSurv.

### 3.2. Cox Proportional Hazards Model

The Cox model was used as the main interpretable survival analysis method in this study and was fitted in Python using the lifelines package. It is widely applied in medical research because it can handle censored data and include multiple clinical variables simultaneously. The model estimates the hazard function by combining a baseline hazard with the effects of covariates, allowing investigators to evaluate how factors such as age and treatment are associated with survival outcomes [12].

An important assumption of the Cox model is the proportional hazards (PH) assumption, which states that the effect of each covariate remains constant over time [20]. This assumption was assessed using a Grambsch–Therneau-type proportional hazards test based on scaled Schoenfeld residuals, implemented with the proportional_hazard_test function in the lifelines package using rank-transformed time [21,22]. The corresponding *p*-values test the null hypothesis that the proportional hazards assumption holds for each covariate.

The Cox model estimates HRs, which are readily interpretable in clinical research. An HR greater than 1 indicates a higher hazard, whereas an HR less than 1 indicates a lower hazard relative to the reference group. These estimates help describe associations between clinical factors and patient survival.

### 3.3. DeepSurv Model

DeepSurv, a deep learning-based survival model, was implemented in PyTorch to improve predictive performance. It extends the Cox model by replacing the linear predictor with a neural network, allowing the model to capture nonlinear relationships and interactions between variables [13]. The model is trained using a loss function based on the Cox partial likelihood, which preserves the ability to handle censored data. Compared with traditional Cox regression, DeepSurv provides more flexible modeling for high-dimensional and complex clinical data, making it a powerful approach for survival prediction in medical research.

The network consisted of two hidden fully connected layers with 64 and 32 neurons, respectively. ReLU activation functions were used after each hidden layer, and dropout with a rate of 0.30 was applied after the first hidden layer to reduce overfitting [23]. The output layer contained a single neuron representing the predicted log-risk score. The model was optimized using the Adam optimizer with a learning rate of 0.001 for 200 epochs. Full-batch training was used, and no weight decay was applied. The Cox partial-likelihood loss was computed on the training set. With the final encoded feature set, the neural network contained approximately 2881 trainable parameters, indicating a relatively small model that is feasible for ordinary clinical research computing environments.

### 3.4. Model Evaluation

Model performance was evaluated using the C-index, which measures concordance between predicted risks and observed survival outcomes. The C-index is one of the most commonly used metrics in survival analysis because it focuses on the model’s ability to correctly rank patients by risk [24]. In this context, it evaluates whether patients with higher predicted risk tend to experience the event earlier than those with lower predicted risk.

The C-index ranges from 0.5 to 1. A value of 0.5 indicates performance no better than random ranking, whereas values closer to 1 indicate stronger discrimination. Therefore, a higher C-index reflects better model discrimination.

In this work, all models were first evaluated on a held-out test set to ensure a fair comparison. To quantify uncertainty in the primary model comparison, a paired bootstrap confidence interval was calculated for the difference in C-index between DeepSurv and Cox on the held-out test set. In response to concerns about reliance on a single random split, five-fold cross-validation was additionally conducted. To prevent data leakage, preprocessing steps, including standardization and categorical encoding, were fitted within each training fold and then applied to the corresponding validation fold. External validation on an independent cohort remains necessary before clinical deployment.

### 3.5. Treatment Recommendation Framework

To extend survival prediction toward clinical decision support, a personalized treatment recommendation framework was developed based on the trained DeepSurv model. In this framework, treatment modality was included as one of the input variables of the model together with other patient characteristics including age, smoking-related variables, sex, and tumor stage.

For each patient, multiple simulated treatment scenarios were generated by changing only the treatment modality variable while keeping all other clinical covariates unchanged. Specifically, each patient was assigned different treatment options, including RT alone, ChemoRT, and RT + EGFRI, one at a time. This approach allowed the model to estimate the relative survival risk under different treatment strategies for the same patient profile.

The trained DeepSurv model was then used to predict a relative risk score for each simulated treatment scenario. The risk score produced by DeepSurv represents the patient’s hazard relative to a baseline risk level, where lower values indicate lower predicted risk. For each patient, the treatment associated with the lowest predicted relative risk was selected as the recommended treatment option.

After generating recommendations, the predicted optimal treatments were compared with the actual treatments recorded in the dataset. This comparison was used to evaluate the consistency between model-based recommendations and real clinical decisions.

The recommendation algorithm can be summarized as follows:

1. Fit the DeepSurv model using the training data and observed treatment modality.
2. For each patient, create three copies of the covariate vector corresponding to RT alone, ChemoRT, and RT + EGFRI.
3. Keep age, sex, smoking variables, and tumor stage fixed across the three copies.
4. Predict the relative risk score for each hypothetical treatment scenario.
5. Select the treatment with the lowest predicted relative risk as the model-recommended option.

Because treatment assignment in the original dataset was observational rather than randomized, this procedure estimates model-predicted risk under hypothetical treatment coding. It should not be interpreted as estimating causal treatment effects without additional causal adjustment.

## 4. Results

### 4.1. Analytical Cohort and Baseline Characteristics

After preprocessing and variable selection, the final analytical dataset included 3266 patients. Starting from the original 3346 patients, three patients in the postoperative RT alone group were excluded because this group was too small for stable treatment-specific modeling. Additional exclusions occurred during stage harmonization and removal of unknown or missing stage records. The final baseline predictors used for model development were age, smoking pack-years, sex, smoking status, merged tumor stage, and treatment modality. Survival outcomes were defined using Length FU and Status.

The final treatment groups were imbalanced. A total of 1792 patients (54.9%) received radiotherapy alone, 1402 (42.9%) received chemoradiotherapy, and 72 (2.2%) received radiotherapy plus EGFR inhibitor. No resampling or class-balancing strategy was applied in the primary analysis because the outcome was modeled as time-to-event survival rather than treatment-class classification. However, this imbalance is important when interpreting treatment-specific predictions, particularly for the RT + EGFRI scenario.

Baseline demographic and clinical characteristics of the study population are summarized by treatment group in Table 3. The descriptive results indicate clinically meaningful differences across treatment groups. Patients receiving RT alone or RT + EGFRI tended to be older than those receiving ChemoRT, whereas smoking exposure and disease site distributions also differed across groups. Stage distribution was particularly imbalanced. Most patients in the ChemoRT and RT + EGFRI groups had stage IV disease, while the RT-alone group included a larger proportion of patients with earlier-stage disease. These baseline differences should be considered when interpreting unadjusted survival comparisons and treatment-specific model predictions.

### 4.2. Kaplan–Meier Curves

**Figure 3:**
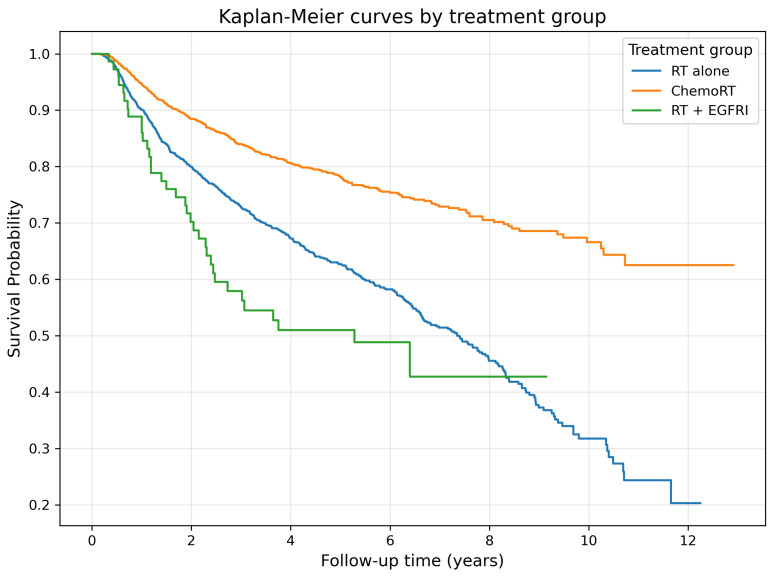
Kaplan–Meier curves by treatment group.

Kaplan–Meier (KM) survival curves were used to estimate overall survival probabilities across treatment groups. As a non-parametric method, KM estimation does not require strict assumptions about the survival-time distribution and provides a descriptive summary of survival outcomes over time. The corresponding survival curves are presented in Figure 3. The survival curves visually suggest differences in survival outcomes among the three treatment groups. The ChemoRT group appeared to have the highest survival probability over much of the follow-up period, followed by the RT alone group, whereas the RT + EGFRI group appeared to have lower survival.

A log-rank test showed evidence of differences among the treatment groups (test statistic = 134.13, *p* < 0.001). However, because treatment assignment was not randomized, this result should be interpreted as an unadjusted group comparison rather than a causal treatment-effect estimate.

### 4.3. Cox Proportional Hazards Model Results

#### 4.3.1. Proportional Hazards Assumption

To assess the validity of the Cox model, the PH assumption was evaluated using a scaled Schoenfeld residual-based test, with the detailed results presented in Table 4. Among all variables, Stage IV disease showed the strongest evidence of deviation from the PH assumption, with a test statistic of 33.20 and p < 0.005, indicating that its association with survival may vary over time. Stage III disease also showed evidence of deviation, with a test statistic of 13.84 and *p* < 0.005.

In contrast, age showed a milder deviation from the PH assumption, with a test statistic of 5.68 and a *p*-value of 0.02. Evidence against the PH assumption was weaker for male, with a test statistic of 2.65 and a *p*-value of 0.10. The smoking status-related variables and Stage II disease showed minimal evidence of deviation. The test statistics for smoking status subgroups were very low (0.01–0.15), with corresponding p-values ranging from 0.70 to 0.91, suggesting that their effects on survival were approximately stable over time.

The results indicated violations of the PH assumption for age and, more importantly, for advanced tumor stage subgroups (Stage III and Stage IV). These findings suggest that the prognostic effect of advanced stage may vary over follow-up time. The Cox model was therefore retained primarily as an interpretable baseline model, while the corresponding hazard ratios were interpreted as average associations over the observed follow-up period. Future analyses should consider time-varying coefficients, stratified Cox models, or flexible survival models to address non-proportional hazards more directly [20,25].

#### 4.3.2. Cox Regression Results

**Figure 4:**
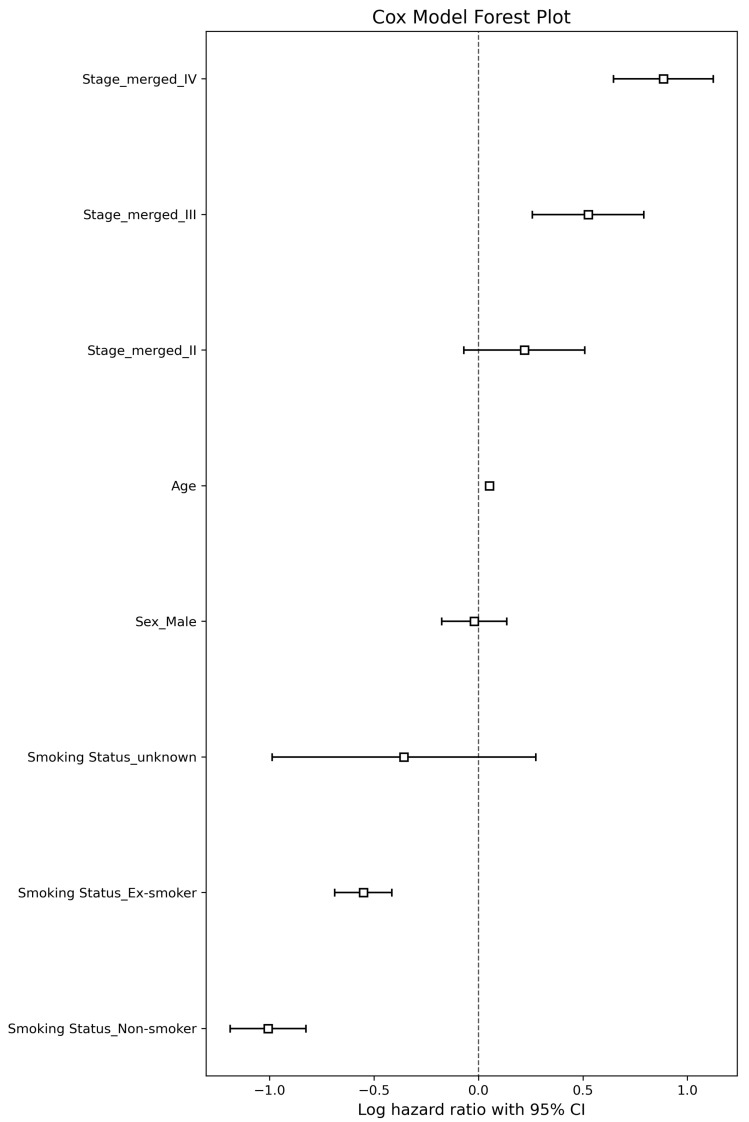
Forest plot.

Table 5 summarizes the HRs, corresponding 95% CIs, and *p*-values estimated from the Cox model. These results quantify associations between clinical variables and patient survival outcomes. The Cox model results are further visualized using a forest plot, shown in Figure 4. Tumor stage was referenced to Stage I disease. Sex was referenced to female patients, and smoking status was referenced to current smokers.

Tumor stage showed a notable association with survival outcomes. Compared with Stage I disease, Stage III disease was associated with increased mortality risk (HR = 1.69, 95% CI: 1.29–2.21), and Stage IV disease was associated with an even higher risk (HR = 2.42, 95% CI: 1.91–3.07). Stage II disease was not statistically significant at the conventional 0.05 level (HR = 1.24, 95% CI: 0.93–1.66). Smoking status also played an important role. Compared with current smokers, former smokers and non-smokers had lower estimated hazards, whereas unknown smoking status was not statistically significant. Age was associated with a higher hazard, with each additional year corresponding to an estimated 5% increase in mortality hazard. Sex was not statistically significant in this multivariable model.

Clinically, these findings are consistent with the recognized prognostic roles of tumor stage and age in head and neck cancer, and the reduced hazards observed among former and non-smokers support the survival benefit of smoking cessation. The non-significant predictors, including sex, Stage II disease, and unknown smoking status, should not be interpreted as clinically irrelevant. Their lack of statistical significance may reflect limited statistical power, modest effect sizes, or mediation through other covariates. These variables were retained because they are clinically relevant baseline characteristics and help preserve a consistent adjustment set. In particular, the unknown smoking category was retained to avoid potential bias associated with complete-case exclusion. Because only a small number of clinically selected predictors were included in the model, substantial multicollinearity was not expected. Nevertheless, variance inflation factor (VIF) analysis was performed to verify this assumption, and all predictor variables had VIF values below 3, indicating no evidence of problematic multicollinearity.

Overall, tumor stage, age, and smoking status were the main prognostic factors identified by the Cox model.

### 4.4. DeepSurv Model Results

The DeepSurv model used the final preprocessed clinical variables, including age, smoking pack-years, sex, smoking status, merged tumor stage, and treatment modality, as input features. Continuous variables were standardized, while categorical variables were transformed using one-hot encoding before training.

**Figure 5:**
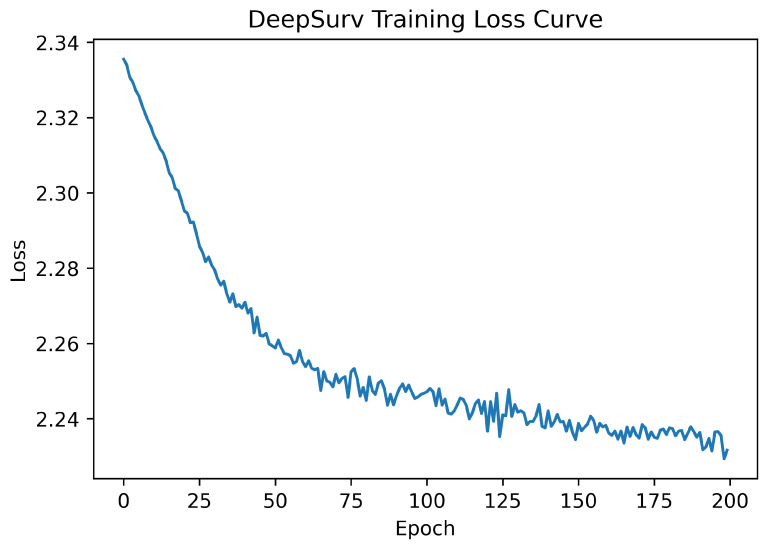
DeepSurv training loss curve.

The dataset was randomly split into a training set (80%) and a test set (20%). The DeepSurv model was trained on the training set using a Cox partial-likelihood loss function and the Adam optimizer for 200 epochs. During training, the model learned to assign higher risk scores to patients who experienced events earlier and lower risk scores to those with longer survival times. The test set was used only for final model evaluation. Figure 5 illustrates the training loss curve of the DeepSurv model. The loss gradually decreased and stabilized during training, suggesting that the optimization process converged without severe instability.

The proposed DeepSurv model contained 2881 trainable parameters and required approximately 0.2 s for model training, whereas the average inference time for a single patient was below 0.001 ms. These results indicate that the computational burden of the proposed model is low and is unlikely to limit future clinical deployment. Instead, practical implementation will primarily depend on external validation, integration into clinical workflows, and regulatory considerations.

### 4.5. Model Performance Comparison

The models evaluated in this study included the traditional Cox proportional hazards model and the deep learning-based DeepSurv model. Predictive performance was compared using the held-out test-set C-index and five-fold cross-validation. The uncertainty of the primary held-out comparison was further assessed using a paired bootstrap confidence interval for the C-index difference between DeepSurv and Cox. Table 6 summarizes the held-out C-index, the paired bootstrap difference relative to Cox, and the five-fold cross-validation results.

The Cox model had a held-out test C-index of 0.684, whereas the DeepSurv model had a held-out test C-index of 0.695. The absolute difference was 0.011, indicating that DeepSurv provided only a modest numerical improvement over the interpretable Cox baseline. The paired bootstrap 95% CI for the difference ranged from −0.010 to 0.030; because this interval included zero, the difference in C-index between the two models was not statistically significant. Five-fold cross-validation showed the same directional pattern, with mean C-index values of 0.707 for DeepSurv and 0.688 for Cox.

Table 7 presents the benchmark results for additional state-of-the-art survival machine-learning models evaluated using the same held-out test set. These benchmark methods were selected because they are widely used and well-established machine-learning approaches for survival analysis, thereby providing appropriate reference models for evaluating the proposed DeepSurv framework. Among these models, random survival forest had the highest held-out test C-index (0.687), followed by gradient boosting survival (0.679), LightGBM survival (0.675), and XGBoost survival (0.675). These benchmark models did not exceed the held-out C-index observed for DeepSurv in this dataset.

Table 8 summarizes the feature ablation analysis of the proposed DeepSurv framework, in which progressively expanded predictor sets were evaluated using the held-out test-set C-index. The analysis showed that tumor stage contributed the largest improvement in model discrimination, increasing the C-index from 0.654 to 0.688, whereas treatment modality provided an additional, albeit more modest, improvement. The full model including treatment modality achieved the highest held-out C-index. These results support the contribution of disease stage and treatment information to the predictive framework.

### 4.6. DeepSurv-Based Treatment Recommendation

A personalized treatment recommendation framework was developed using the trained DeepSurv model to translate the model’s predictive capabilities into practical clinical decision support.

To demonstrate the application of the personalized treatment recommendation framework, one patient was first selected as an illustrative case study. The corresponding treatment-specific risk values and model-recommended treatment are shown in Table 9.

The same recommendation logic was then applied to the first 20 patients in the dataset. Their model-recommended treatments are shown in Table 10.

The model was subsequently applied to all patients in the dataset to summarize the overall distribution of model-recommended treatments.

**Figure 6:**
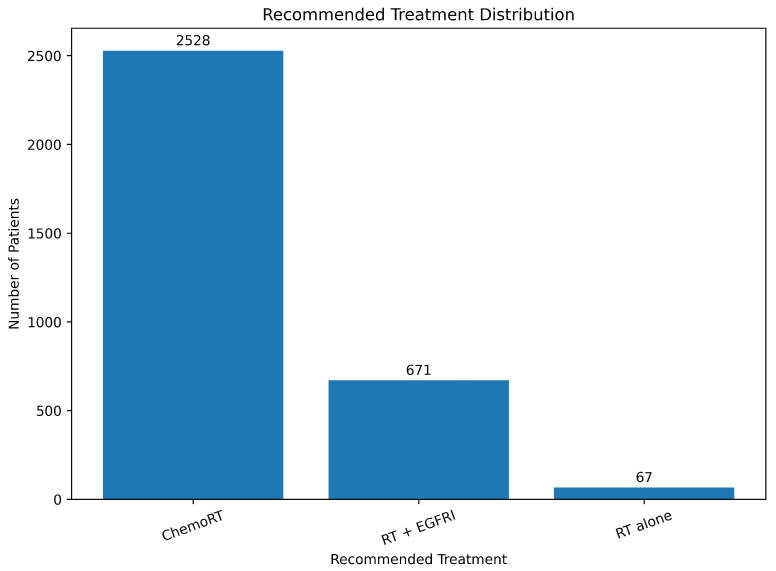
Model-recommended treatment distribution for all patients.

Figure 6 shows the distribution of recommended treatment plans among all patients using the original DeepSurv recommendation output. ChemoRT was the most frequently recommended treatment, followed by RT + EGFRI, whereas RT alone was recommended for the smallest number of patients. These results indicate that the model generated different treatment choices according to patient-level covariate profiles rather than assigning the same treatment to all patients.

**Figure 7:**
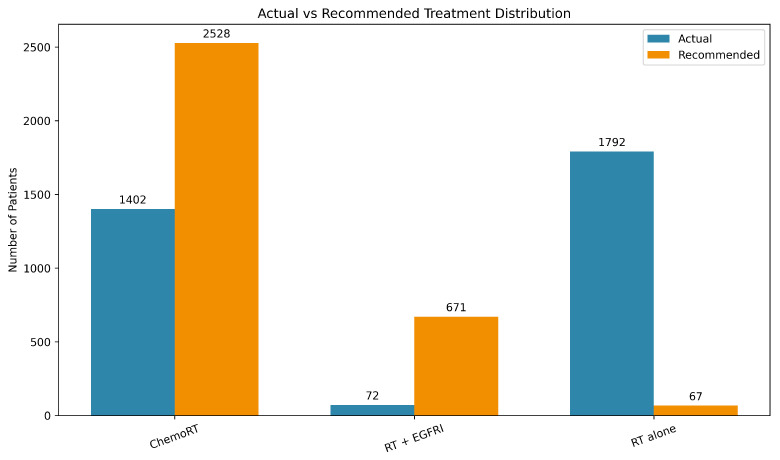
Comparison of actual and model-recommended treatment distributions.

To further examine this difference, Figure 7 compares the distribution of treatments actually received by patients with the treatments recommended by the model. The grouped bar chart displays the number of patients in each treatment category and makes the differences between observed and recommended treatment distributions easier to interpret.

Specifically, the actual treatment data show that radiotherapy alone was the most commonly used treatment, followed by chemoradiotherapy, whereas radiotherapy combined with EGFRI was used least often. In contrast, the model-recommended treatments favored ChemoRT most frequently, followed by RT + EGFRI and then RT alone. It should be noted that the model’s recommendations are based on predictive modeling rather than causal inference. The discrepancy between observed and recommended treatments should be interpreted as a model-based difference in predicted risk under hypothetical treatment scenarios rather than evidence that the model recommendation is clinically superior.

Real-world treatment decisions are shaped by disease extent, age, frailty, comorbidities, organ function, toxicity concerns, patient preferences, socioeconomic conditions, travel burden, and healthcare access. These factors are not fully captured in the structured variables used by the model. In addition, large clinical datasets may underrepresent frail patients and patients facing barriers to care, which can lead to selection bias and reduce the generalizability of deep-learning-based recommendations. Therefore, in clinical practice, such recommendations should be considered only as decision-support information and should be integrated with clinician judgment, patient preferences, toxicity assessment, and multidisciplinary review.

## 5. Discussion

In the held-out 80%/20% evaluation, the DeepSurv model had a numerically higher C-index than the Cox model (0.695 vs. 0.684). However, the paired bootstrap 95% CI for the C-index difference included zero, indicating that the difference in discrimination between the two models was not statistically significant. Five-fold cross-validation produced a similar directional pattern, with mean C-index values of 0.707 for DeepSurv and 0.688 for Cox. These findings suggest that DeepSurv may offer additional modeling flexibility, but the observed gain over the interpretable Cox baseline was small and should not be interpreted as clear evidence of superior predictive performance. Therefore, the Cox model remained a competitive baseline due to its interpretability and simplicity.

The comparison with existing methods reported in recent literature should be interpreted qualitatively rather than as a direct numerical benchmark, as substantial heterogeneity exists across studies in cohort composition, endpoints, feature sets, treatment settings, modeling objectives, and validation protocols [4,5,7,8].

The Cox model identified tumor stage as one of the strongest predictors of survival, with more advanced stages associated with higher hazard rates. This finding is consistent with established clinical knowledge and reflects the importance of disease progression in treatment planning [26]. Smoking-related variables were also associated with survival outcomes, which is consistent with previous studies in patients with head and neck cancer [27,28]. In comparison, age and sex showed relatively weaker effects. Overall, the consistency between the model findings and existing clinical evidence supports the validity of the analysis.

A notable finding was that the treatments recommended by the model often differed from those selected in clinical practice. This discrepancy is expected because the model only considers variables available in the dataset and focuses on predicted survival risk, whereas real clinical decisions are influenced by additional factors such as comorbidities, treatment toxicity, physician experience, patient preferences, social support, and healthcare access [29,30]. Large observational datasets may also underrepresent frail patients and individuals facing socioeconomic or access-related barriers, which can introduce selection bias and affect the generalizability of model-based recommendations. This issue is consistent with broader concerns about inclusivity and eligibility-related bias in oncology research [31]. Furthermore, the recommendation framework is based on simulated treatment scenarios in which treatment modality is changed while all other patient characteristics remain fixed. Therefore, the recommendations should be interpreted as estimated risks under hypothetical treatment options rather than causal treatment effects.

Several limitations should be acknowledged. First, the dataset was obtained from a single institution, which may limit the generalizability of the findings to other populations and clinical settings [15]. Second, the treatment groups were highly imbalanced, particularly for RT + EGFRI, which may reduce the reliability of predictions for this treatment scenario. Third, some treatment subgroups with very small sample sizes were excluded to improve model stability, potentially reducing the completeness of the analysis. Fourth, violations of the proportional hazards assumption were observed for age and advanced tumor stage, which may affect the interpretation of Cox-model hazard ratios. Fifth, although five-fold cross-validation and a paired bootstrap confidence interval for the C-index difference were added, no independent external validation cohort was available. Sixth, excluding variables with high missingness instead of using multiple imputation reduces imputation-related uncertainty but may introduce selection bias if patients with missing data differ systematically from the analyzed cohort. Finally, the treatment recommendation framework is not based on causal inference and therefore cannot establish true treatment effects.

Future research should incorporate additional factors that influence treatment decisions, including treatment tolerance, comorbidities, frailty, toxicity risk, social determinants of health, and patient preferences. More advanced approaches, such as causal inference methods, may help reduce bias arising from observational data and improve estimation of treatment effects [32]. Integrating other data sources, such as medical imaging or genomic information, may further improve predictive performance and provide a more comprehensive representation of patient status [23]. External validation using datasets from multiple institutions is necessary to evaluate model generalizability [16,25]. Although additional survival machine-learning benchmarks were included, future studies should further evaluate these methods with prespecified hyperparameter tuning, calibration assessment, and external validation. Finally, improving the interpretability of deep learning models and deployment requirements may increase their acceptance and usefulness in clinical practice [15].

## 6. Conclusions

This study developed an interpretable Cox-model baseline and a DeepSurv-based survival prediction framework for patients with head and neck cancer using structured clinical variables from the RADCURE dataset. Using the same held-out 80%/20% evaluation split, DeepSurv achieved a C-index of 0.695, compared with 0.684 for the Cox model. However, the paired bootstrap confidence interval for the C-index difference included zero, indicating that the difference was not statistically significant. Five-fold cross-validation showed the same directional pattern, but the overall improvement remained modest. Tumor stage, age, and smoking status were the most important prognostic factors identified in the Cox analysis.

The proposed recommendation framework demonstrates how a survival model can generate patient-specific predicted risks under alternative treatment scenarios. However, these recommendations should be interpreted as decision-support estimates rather than causal evidence of treatment benefit. The small performance gain of DeepSurv, the imbalance of treatment groups, the violation of the proportional hazards assumption for advanced stage, and the absence of external validation all limit the strength of the current conclusions.

For clinicians, the main implication is that survival-based tools may help organize prognostic information and identify cases where treatment options merit multidisciplinary discussion. For future research, the priorities are external validation in independent cohorts, causal methods for treatment-effect estimation, and inclusion of clinically important variables such as comorbidities, frailty, toxicity risk, and patient preferences.

## Figures and Tables

**Table 1:** Description of the original RADCURE clinical variables.

Original Variable	Description	Type or Coding/Categories
patient_id	Randomly assigned anonymized patient identifier.	Identifier.
Age	Patient age.	Continuous, years.
Sex	Patient sex.	Female; Male.
ECOG PS	Eastern Cooperative Oncology Group performance status, reflecting functional status at baseline.	ECOG 0; ECOG 0–1; ECOG 1; ECOG 2; ECOG 3; ECOG 4; ECOG-Pt 0; ECOG-Pt 1; ECOG-Pt 2; Unknown.
Smoking PY	Smoking exposure.	Continuous, pack-years.
Smoking Status	Smoking status at first consultation.	Current; Ex-smoker; Non-smoker; unknown.
Ds Site	Primary cancer site.	Oropharynx; Larynx; Hypopharynx; Nasopharynx; Lip & Oral Cavity; nasal cavity; Nasal Cavity; Paranasal Sinus; Skin; Salivary Glands; Sarcoma; Esophagus/esophagus; Paraganglioma; Orbit; Lacrimal gland; benign tumor; Other; Unknown.
Subsite	Primary cancer subsite.	High-cardinality categorical variable with 63 observed labels, including Glottis, Tonsil, Tonsillar Fossa, Base of Tongue, Soft Palate, Pyriform Sinus, Supraglottis, Tongue, Nasal Cavity, and others.
T	AJCC 7th edition T category.	T0; T1; T1a; T1b; T1 (2); T2; T2a; T2b; T2 (2); T3; T3 (2); T4; T4a; T4b; Tis; TX; rT0.
N	AJCC 7th edition N category.	N0; N1; N2; N2a; N2b; N2c; N3; N3a; N3b; NX.
M	AJCC 7th edition M category.	M0; M1; MX.
Stage	AJCC 7th edition stage group.	0; I; IB; II; IIA; IIB; III; IIIA; IIIC; IV; IVA; IVB; IVC; X.
Path	Pathologic diagnosis or histology type.	High-cardinality categorical variable with 41 observed labels, including Squamous Cell Carcinoma, nasopharyngeal carcinoma subtypes, Adenoid Cystic, Adenocarcinoma, Sarcoma subtypes, Neuroendocrine carcinoma, and others.
HPV	Tumor HPV status determined by p16 immunohistochemistry with or without HPV DNA PCR.	Yes, positive; Yes, Negative; blank = no data available.
Tx Modality	Treatment modality indicating how surgery, radiotherapy, and chemotherapy were combined.	RT alone; ChemoRT; RT + EGFRI; Postop RT alone.
Chemo	Indicator of concurrent chemotherapy.	Yes; none.
RT Start	Date of first radiotherapy fraction.	Offset date.
Dose	Total radiotherapy dose delivered.	Continuous, Gy.
Fx	Number of radiotherapy fractions delivered.	Count.
Last FU	Most recent contact date at which clinical outcomes were updated, or date of death.	Offset date.
Status	Vital status at last contact.	Alive; Dead.
Length FU	Follow-up duration from diagnosis to last contact.	Continuous, years.
Date of Death	Date of death.	Offset date; applicable to patients who died.
Cause of Death	Cause of death, if applicable.	Categorical variable with 24 observed labels, including Index Cancer, Other Cancer, Other Cause, Unknown, and more specific treatment- or cancer-related causes.
Local	Local relapse indicator.	Yes; Persistent; Possible; blank = no recorded local relapse in the source field.
Date Local	Date of local relapse, if applicable.	Offset date.
Regional	Regional relapse indicator.	Yes; Persistent; Possible; blank = no recorded regional relapse in the source field.
Date Regional	Date of regional relapse, if applicable.	Offset date.
Distant	Distant metastasis indicator.	Yes; Persistent; Possible; blank = no recorded distant metastasis in the source field.
Date Distant	Date of distant metastasis, if applicable.	Offset date.
2nd Ca	Second cancer diagnosis indicator.	Y; S (suspicious); blank = no recorded second cancer in the source field.
Date 2nd Ca	Date of second cancer diagnosis, if applicable.	Offset date.
RADCURE-challenge	RADCURE challenge partition indicator.	training; test; 0 = not included in the RADCURE challenge subset.
ContrastEnhanced	Indicator of contrast-enhanced imaging.	1 = contrast-enhanced; 0 = regular/non-enhanced.

**Table 2:** Missing-data rates in the original RADCURE clinical dataset.

Variable	Missing, n	Missing, %
ECOG performance status	1	0.0
Smoking pack-years	5	0.1
Subsite	374	11.2
T stage	12	0.4
N stage	13	0.4
M stage	14	0.4
Overall stage	27	0.8
HPV status	1629	48.7
Date of death	2288	68.4
Cause of death	2294	68.6
Local relapse	2966	88.6
Date of local relapse	2966	88.6
Regional relapse	3157	94.4
Date of regional relapse	3157	94.4
Distant metastasis	2933	87.7
Date of distant metastasis	2933	87.7
Second cancer	2905	86.8
Date of second cancer	2907	86.9

**Table 3:** Baseline characteristics by treatment group.

Characteristic	RT Alone	ChemoRT	RT + EGFRI
Patients, n	1792	1402	72
Age, years			
Mean ± SD	66.7 ± 11.1	56.0 ± 9.3	70.2 ± 8.1
Median (IQR)	67.4 (59.1–75.0)	56.8 (50.5–62.6)	71.9 (68.4–74.4)
Smoking pack-years			
Mean ± SD	30.3 ± 25.6	18.2 ± 20.8	25.8 ± 22.4
Median (IQR)	30.0 (6.0–50.0)	11.0 (0.0–30.0)	20.0 (0.0–46.2)
Sex, n (%)			
Male	1427 (79.6)	1116 (79.6)	61 (84.7)
Female	365 (20.4)	286 (20.4)	11 (15.3)
ECOG performance status, n (%)			
0–1	1578 (88.1)	1338 (95.4)	69 (95.8)
2–5	193 (10.8)	56 (4.0)	3 (4.2)
Unknown	21 (1.2)	8 (0.6)	0 (0.0)
Smoking status, n (%)			
Ex-smoker	747 (41.7)	476 (34.0)	34 (47.2)
Current	678 (37.8)	423 (30.2)	17 (23.6)
Non-smoker	347 (19.4)	484 (34.5)	21 (29.2)
Unknown	20 (1.1)	19 (1.4)	0 (0.0)
Disease site, n (%)			
Larynx	731 (40.8)	95 (6.8)	9 (12.5)
Oropharynx	678 (37.8)	774 (55.2)	48 (66.7)
Hypopharynx	94 (5.2)	60 (4.3)	7 (9.7)
Lip and oral cavity	71 (4.0)	21 (1.5)	5 (6.9)
Nasopharynx	43 (2.4)	312 (22.3)	0 (0.0)
Other or unknown	175 (9.8)	140 (10.0)	3 (4.2)
Stage, n (%)			
I	350 (19.5)	2 (0.1)	0 (0.0)
II	356 (19.9)	47 (3.4)	0 (0.0)
III	371 (20.7)	225 (16.0)	12 (16.7)
IV	715 (39.9)	1128 (80.5)	60 (83.3)
Pathology, n (%)			
Squamous cell carcinoma	1696 (94.7)	1067 (76.1)	72 (100.0)
Nasopharyngeal carcinoma	43 (2.4)	311 (22.2)	0 (0.0)
Other histotypes	49 (2.9)	22 (1.7)	0 (0.0)

Note: Values are presented as mean ±SD, median (IQR), or n(%). Percentages are column percentages within each treatment group. RT = radiotherapy; ChemoRT = chemoradiotherapy; EGFRI = epidermal growth factor receptor inhibitor; ECOG = Eastern Cooperative Oncology Group; IQR = interquartile range; SD = standard deviation.

**Table 4:** Proportional hazards assumption tests.

Covariate	Test Statistic	*p*-Value
Age, per year	5.68	0.02
Male	2.65	0.10
Former smoker	0.15	0.70
Never smoker	0.06	0.81
Unknown smoking status	0.01	0.91
Stage II	0.90	0.34
Stage III	13.84	<0.005
Stage IV	33.20	<0.005

Note: The proportional hazards assumption was assessed using scaled Schoenfeld residuals. Reference categories for indicator variables were female, current smoker, and Stage I disease.

**Table 5:** Cox regression analysis of survival outcomes.

Covariate	HR	95% CI	*p*-Value
Age, per year	1.05	1.05–1.06	<0.005
Male	0.98	0.84–1.14	0.79
Former smoker	0.58	0.50–0.66	<0.005
Never smoker	0.36	0.30–0.44	<0.005
Unknown smoking status	0.70	0.37–1.32	0.27
Stage II	1.24	0.93–1.66	0.14
Stage III	1.69	1.29–2.21	<0.005
Stage IV	2.42	1.91–3.07	<0.005

Note: Hazard ratios were estimated from the multivariable Cox proportional hazards model. Reference categories were female, current smoker, and Stage I disease. CI = confidence interval; HR = hazard ratio.

**Table 6:** Model performance comparison based on held-out test-set C-index and five-fold cross-validation.

Model	Held-Out Test C-Index	Difference vs. Cox (95% CI)	Five-Fold CV C-Index, Mean (SD)
Cox	0.684	Reference	0.688 (0.010)
DeepSurv	0.695	0.011 (−0.010 to 0.030)	0.707 (0.010)

Note: The difference was calculated as the DeepSurv C-index minus the Cox C-index on the held-out test set. The 95% CI for the difference was estimated using paired bootstrap resampling of the held-out test set. Cross-validation preprocessing steps were fitted within each training fold to reduce data leakage. CI = confidence interval; CV = cross-validation; SD = standard deviation.

**Table 7:** Benchmark comparison with state-of-the-art survival machine-learning models.

Model	Held-Out Test C-Index
Random survival forest	0.687
XGBoost survival	0.675
Gradient boosting survival	0.679
LightGBM survival	0.675

Note: All benchmark models were trained and evaluated using the same training and test sets as the Cox and DeepSurv held-out analyses.

**Table 8:** Feature ablation analysis of the proposed DeepSurv framework.

DeepSurv Feature Set	Held-Out C-Index
Age + smoking variables	0.653
Age + smoking variables + sex	0.654
Age + smoking variables + sex + stage	0.688
Full model including treatment modality	0.695

**Table 9:** Treatment recommendation for one illustrative patient.

Treatment Scenario	Relative Risk
Chemoradiotherapy	0.81
Radiotherapy + EGFRI	1.62
Radiotherapy alone	1.27
Recommended treatment: Chemoradiotherapy	

**Table 10:** Treatment recommendations for the first 20 patients.

Patient No.	Recommended Treatment
1	Chemoradiotherapy
2	Radiotherapy alone
3	Chemoradiotherapy
4	Chemoradiotherapy
5	Chemoradiotherapy
6	Chemoradiotherapy
7	Chemoradiotherapy
8	Chemoradiotherapy
9	Chemoradiotherapy
10	Chemoradiotherapy
11	Chemoradiotherapy
12	Chemoradiotherapy
13	Chemoradiotherapy
14	Radiotherapy alone
15	Chemoradiotherapy
16	Chemoradiotherapy
17	Chemoradiotherapy
18	Chemoradiotherapy
19	Chemoradiotherapy
20	Chemoradiotherapy

## Data Availability

The original data used in this study are openly available in The Cancer Imaging Archive (TCIA) at https://doi.org/10.7937/J47W-NM11, reference number RADCURE.
